# Antiretroviral Therapy-Associated Weight Gain in Mexico, a Country Prone to Comorbidities

**DOI:** 10.34172/ijhpm.9004

**Published:** 2025-08-18

**Authors:** Andrés Tapia-Maltos, Brenda Crabtree-Ramírez

**Affiliations:** ^1^Plan de Estudios Combinados en Medicina (PECEM), Facultad de Medicina, Universidad Nacional Autónoma de México (UNAM), Mexico City, Mexico.; ^2^Departamento de Infectología, Instituto Nacional de Ciencias Médicas y Nutrición Salvador Zubirán, Mexico City, Mexico.

## Introduction

 Advancements in antiretroviral therapy (ART) have drastically improved the quality of life for people with human immunodeficiency virus (PWH). Modern ART is more efficient at achieving both virological suppression and immunological reconstitution and at decreasing the emergence of resistance to treatment.^1^ Even though the progress in treatment has transformed HIV infection into a controllable condition, adverse events caused by ART remain a matter of concern. From zidovudine-induced lipodystrophy to lucid dreaming and insomnia associated with efavirenz (EFV), quality of life of PWH is also impacted by the adverse events ART may cause, particularly in aging populations.^2,3^ Second-generation integrase-strand transfer inhibitors (INSTIs) have proven to be one of the most efficient and tolerable antiretroviral classes, making them the first-line treatment for HIV infection in guidelines worldwide. However, INSTIs are not free of undesired effects, among which weight gain has become an important subject of research.^4^ Our approach to collect relevant evidence for this matter includes a literature search in PubMed using keywords (“integrase inhibitors”[MeSH Terms] OR INSTI) AND ((“weight gain”[MeSH Terms] OR “body weight” OR “BMI”) AND (“HIV”[MeSH Terms] OR HIV)) and filters (observational studies, clinical trials, humans, 2015-2024), as well as publications referenced in current Mexican ART guidelines. We selected studies that offered robust data on differences between ART options, risk factors for weight gain, and their association with metabolic comorbidities. Regarding epidemiological information, we looked for primary sources that presented data on the prevalence of metabolic comorbidities published by national public health institutions, focusing on those related to metabolic syndrome.

## How INSTIs Relate to Body Weight

 While the initiation of an INSTI and tenofovir alafenamide (TAF) has been found to be associated with weight gain, the discontinuation of EFV and tenofovir disoproxil (TDF) may also play a role in this phenomenon.^4^ An observational study with data from the North American AIDS Cohort Collaboration on Research and Design concluded that combinations which include INSTIs were associated with a greater increase in weight compared to protease inhibitors (PIs) and non-nucleoside reverse-transcriptase inhibitors (NNRTIs)-based ART.^5^ Furthermore, in randomized clinical trials, the ADVANCE study compared two ART regimens that contained the INSTI dolutegravir (DTG), DTG/TAF/Emtricitabine (FTC) and DTG/TDF/FTC, with EFV/TDF/FTC.^6^ The ART combination that included TAF induced greater weight gain than the ones with TDF. Patients who used a regimen containing DTG also presented a significant weight gain compared to those on the EFV arm.^6^ Therefore, the degree to which INSTIs contribute to changes in weight may be dependent on the clinical context and ART history of each patient.

 Initiation of ART is expected to induce weight gain in patients with advanced HIV infection, particularly in those with wasting syndrome, a beneficial phenomenon known as “return to health.”^7^ Nevertheless, weight gain is not desirable in all PWH who begin ART.^7,8^ In people with early stages of HIV infection and in those who have attained virological suppression through ART, beginning or switching to a combination that includes an INSTI may be cause of concern for involuntary weight gain.^8^ Moreover, in those who already have overweight, obesity, or another predisposition for metabolic disease, starting or switching to an INSTI-based therapy may impose additional preoccupation for weight management and worsening of their condition.^8^ This event does not appear in most patients who use INSTIs, and its presentation may be dependent on non-modifiable factors such as age, sex assigned at birth, race, and ethnicity.^7,8^ Considerable involuntary weight gain may burden patients with interrelated non-communicable conditions which PWH are already at a higher risk of developing than the general population, such as diabetes mellitus, systemic arterial hypertension, dyslipidemia, and cardiovascular disease.^7,8^ As a consequence, given the higher life expectancy that has derived from the development and worldwide availability of ART, metabolic comorbidities have taken a major role as causes of death in PWH.^9^

## Comorbidities’ Situation in Mexico

 Besides having one of the highest childhood obesity rates (35%) in the world, around 75% of the adult Mexican population has either overweight or obesity, a condition that contributes to the development of metabolic comorbidities.^10^ Furthermore, according to the 2022 National Health and Nutrition Survey, the prevalence of diabetes mellitus in Mexican adults is estimated to be around 18%, of which 5% remain unaware of their diagnosis.^11^ Data revolving around the prevalence of systemic arterial hypertension in Mexico vary, but most sources, including the aforementioned 2022 survey, agree it is somewhere between 25%-30% of the adult population, according to the Eight Joint National Committee classification.^12^ Likewise, dyslipidemia prevalence depends on classification, with hypoalphalipoproteinemia, the most prevalent lipid abnormality in Mexico, having been consistently estimated to be present in 55%-60% of adults in these surveys.^13^ Hypertriglyceridemia and hypercholesterolemia affect between 40% and 50% of the adult Mexican population.^13^ Lastly, cardiovascular disease, a condition whose risk factors include diabetes, hypertension, and dyslipidemia, is the leading cause of death in Mexico.^14^ Given that the development of these chronic non-communicable diseases is more likely in those with overweight or obesity, weight management strategies are essential tools in the prevention and control of metabolic comorbidities. Mexico is regarded as an upper-middle-income country, despite substantial social inequality driven by multidimensional poverty, which includes disparities in access to health services.^15,16^ Additionally, the fragmented health system in Mexico hinders the optimization of medical attention, including the early diagnosis and adequate care of non-communicable disease, which represents an enormous challenge. As a consequence, prevention of comorbidities in Mexico has been unsuccessful in the general population, whose vulnerability to these diseases is enhanced by sociocultural and genetic factors.

## Current HIV Treatment Situation in Mexico

 Ever since the evidence of resistance to NNRTIs in the Latin American region, including Mexico, was observed, national and international guidelines have promoted the use of combinations that include INSTIs as first-line treatment for PWH.^17,18^ In Mexico, DTG and bictegravir (BIC) have become the most recommended INSTIs for combined ART. After an agreement done by the Mexican government in 2019 culminated in the massive purchase of BIC/TAF/FTC, this regimen has become the first-line treatment nationwide; since then, Mexico is the only country in the region that has had a massive rollout of this ART combination. Despite their excellent results at attaining an undetectable viral load and less directly harmful adverse events, INSTIs’ association with involuntary weight gain could become a problem for PWH in Mexico, whose social determinants of health and genetic factors, as described above, may have already put them at risk of metabolic comorbidities. Even though the benefit of achieving and maintaining virological suppression with INSTIs is greater than the risk of developing comorbidities associated with clinically significant weight gain, medical practitioners find themselves at a crossroads when it comes to the management of this adverse event. Surveillance of weight and metabolic laboratory values (serum glucose, cholesterol, triglycerides, etc) is crucial in the follow-up of patients who have begun using an INSTI, especially in the first year after initiation.

## What Is There to Be Done?

 Current guidelines in Mexico offer alternatives in ART, whether it be because of toxicity, adverse events, potential interaction with other drugs, or treatment optimization. However, alternatives in current guidelines often represent regimens which are more difficult to tolerate and increase the pill burden on patients, whether they be NNRTIs or PIs. While both national and international guidelines adequately promote the use of combinations that include an INSTI, no recommendations are given in the case of weight gain associated with this class of antiretrovirals.

 Current evidence, however, has not addressed how to properly manage weight changes in PWH who use an INSTI-based ART regimen. For example, the ongoing DEFINE study included virologically suppressed patients who had been on an INSTI-based therapy for at least three years and who had had a weight increase of at least 10% while on treatment. This clinical trial did not find any significant difference in weight change between patients who switched to a boosted PI-based regimen and those who remained on the INSTI-based ART 24 weeks after randomization.^19^ A similar study (P018), in which randomized patients either switched to doravirine/islatravir or remained on BIC/TAF/FTC, did not find any significant difference in weight and body composition changes between both groups after 48 weeks.^20^ Therefore, the management of weight gain in INSTI users may not be as simple as an ART regimen change.

 In the French DoraVIH observational cohort study, a majority of patients who decided to switch to a doravirine-based regime because of tolerability issues did so because of the lack of association between this ART and weight gain.^21^ On the other hand, a small study in Nigeria explored the perspectives of healthcare providers through interviews. They found that excessive weight gain is not regarded as a major concern in this specific population, in part because its deleterious effects may not be seen in the short term and, perhaps even more interestingly, because patients themselves express fear of stigma if they return to an underweight state.^22^ Both of these studies exemplify that ART-driven weight changes have an impact on patient and healthcare provider perspectives. Understanding the complex interplay between patient experiences and real-world clinical decision-making extends beyond mere pharmacological considerations. Patient education, psychological assessment of body perception, and decision-making that combines clinical research evidence with patient-centered priorities and preferences are some of the strategies that policy-makers should include in future guidelines that address this rising issue.

 There is no defined threshold in weight increase that helps to determine if a patient warrants a change in therapy. There are also no established weight conditions, including obesity, that hinder health providers from prescribing an INSTI in the context of naïve population. Even if the weight gain is significant enough to make physicians consider modifying therapy, health providers might find themselves restricted as to what the best next step would be. Balancing between plainly suggesting lifestyle changes or switching to a different ART regimen may not depend on the gained weight alone. Other considerations include availability of alternatives, previous antiretroviral history, and patients’ willingness to switch to another treatment.

## Conclusions

 In addition to the lack of evidence regarding what to do in the context of abnormal weight gain after initiating or switching to INSTI-based regimens, there is also limited information available as to how significantly this adverse event might affect related metabolic surrogate markers in INSTI users. Moreover, the underrepresentation of PWH in Latin America and transgender women in clinical trials imposed a gap of knowledge in this regard. Thus, we call on local governments and private health organizations alike to direct their efforts towards research that studies the relationship between INSTI-associated weight gain and metabolic abnormalities, including surrogate markers such as changes in body composition and energy expenditure and hormonal pathways that may alter appetite, as necessary means to shed light on this problematic. Current studies, particularly clinical trials, have also been limited in assessing dietary and physical activity habits and, therefore, have missed an adequate strategy to overcome this clinical challenge. Through this viewpoint, we hope to have highlighted how consequential tackling this problem could be for countries like Mexico, where INSTIs are used as first-line treatment in a population already vulnerable to comorbidities associated with an excessive weight increase.

## Ethical issues

 Not applicable.

## Conflicts of interest

 Authors declare that they have no conflicts of interest.

## References

[1] Sokhela S, Lalla-Edward S, Siedner MJ, Majam M, Venter WD (2023). Roadmap for achieving universal antiretroviral treatment. Annu Rev PharmacolToxicol.

[2] Guzman N, Vijayan V. HIV-associated lipodystrophy. In: StatPearls. Treasure Island, FL: StatPearls Publishing; 2020. https://www.ncbi.nlm.nih.gov/books/NBK493183/. 29630235

[3] Pau AK, George JM (2014). Antiretroviral therapy: current drugs. Infect Dis Clin North Am.

[4] Wood BR, Huhn GD (2021). Excess weight gain with integrase inhibitors and tenofovir alafenamide: what is the mechanism and does it matter?. Open Forum Infect Dis.

[5] Bourgi K, Jenkins CA, Rebeiro PF (2020). Weight gain among treatment-naïve persons with HIV starting integrase inhibitors compared to non-nucleoside reverse transcriptase inhibitors or protease inhibitors in a large observational cohort in the United States and Canada. J Int AIDS Soc.

[6] Venter WD, Moorhouse M, Sokhela S (2019). Dolutegravir plus two different prodrugs of tenofovir to treat HIV. N Engl J Med.

[7] Kumar S, Samaras K (2018). The impact of weight gain during HIV treatment on risk of pre-diabetes, diabetes mellitus, cardiovascular disease, and mortality. Front Endocrinol (Lausanne).

[8] Venter WD, Serenata C, Vitoria M (2021). What we have learned from antiretroviral treatment optimization efforts over the last 5 years?. AIDS.

[9] MacCann R, Landay AL, Mallon PW (2023). HIV and comorbidities - the importance of gut inflammation and the kynurenine pathway. CurrOpin HIV AIDS.

[10] Lugo G. Obesidad, Epidemia Agudizada en México. Gaceta UNAM; 2021. https://www.gaceta.unam.mx/obesidad-epidemia-agudizada-en-mexico/.

[11] Instituto Mexicano del Seguro Social (IMSS). IMSS Realizó Casi 3.5 Millones de Atenciones de Pacientes Que Viven Con Diabetes Durante. IMSS; 2023. https://imss.gob.mx/prensa/archivo/202311/569#:~:text=Inform%C3%B3%20que%20la%20Encuesta%20Nacional. Accessed May 2, 2024.

[12] Campos-Nonato I, Oviedo-Solís C, Vargas-Meza J (2023). Prevalencia, tratamiento y control de la hipertensión arterial en adultos mexicanos: resultados de la Ensanut 2022. Salud Publica Mex.

[13] Aguilar-Salinas CA. Epidemiología de Las Enfermedades Metabólicas Resultantes de La Malnutrición: El Caso de México. Alimentación y Salud. https://alimentacionysalud.unam.mx/epidemiologia-enfermedades-metabolicas/.

[14] Instituto Nacional de Estadística y Geografía (INEGI). Estadísticas de Defunciones Registradas (EDR). 2023. https://www.inegi.org.mx/app/saladeprensa/noticia.html?id=8911#:~:text=Entre%20enero%20y%20septiembre%20de. Accessed May 2, 2024.

[15] World Bank. Data for Upper Middle Income, Mexico. 2019. https://data.worldbank.org/?locations=MX-XT.

[16] Consejo Nacional de Evaluación de la Política de Desarrollo Social (CONEVAL). Medición de Pobreza. 2022. https://www.coneval.org.mx/Medicion/MP/Paginas/Pobreza_2022.aspx.

[17] Bertagnolio S, Hermans L, Jordan MR (2021). Clinical impact of pretreatment human immunodeficiency virus drug resistance in people initiating nonnucleoside reverse transcriptase inhibitor-containing antiretroviral therapy: a systematic review and meta-analysis. J Infect Dis.

[18] García-Morales C, Ávila-Ríos S. Prevalencia de Resistencia Adquirida a Fármacos Antirretrovirales en El Centro de México. 2020. https://www.gob.mx/cms/uploads/attachment/file/626488/Bolet_n_de_Atenci_n_Integral_de_Personas_con_VIH__Censida.pdf.

[19] Short WR, Ramgopal M, Hagins DP, et al. 1985. A prospective, randomized trial to assess a protease inhibitor–based regimen switch strategy to manage integrase inhibitor–related weight gain. Open Forum Infect Dis 2023;10(Suppl 2):ofad500.112. doi: 10.1093/ofid/ofad500.112. PMC11912975 39230668

[20] McComsey GA, Molina JM, Mills AM, et al. Weight and body composition after switch to doravirine/islatravir (DOR/ISL) 100/0.75mg once daily: week 48 results from 2 randomized active-controlled phase 3 trials, MK8591A-017 (P017) and MK8591A-018 (P018). https://www.natap.org/2023/IAS/IAS_51.htm. Accessed May 2, 2024.

[21] Pourcher V, Robineau O, Parienti JJ (2025). Factors influencing antiretroviral therapy switching in people with virologically suppressed HIV-1: a cross-sectional multicenter study in France. AIDS.

[22] Olawepo JO, Pharr JR, Kabir R, Olutola A (2021). Health care providers’ perceptions about overweight and obesity among people living with human immunodeficiency virus in Nigeria. Qual Health Res.

